# Characterization of the genetic environment of *bla*_ESBL_ genes, integrons and toxin-antitoxin systems identified on large transferrable plasmids in multi-drug resistant *Escherichia coli*

**DOI:** 10.3389/fmicb.2014.00716

**Published:** 2015-01-06

**Authors:** Juan Wang, Roger Stephan, Katrin Zurfluh, Herbert Hächler, Séamus Fanning

**Affiliations:** ^1^UCD Centre for Food Safety, School of Public Health, Physiotherapy and Population Science, University College DublinDublin, Ireland; ^2^Vetsuisse Faculty, Institute for Food Safety and Hygiene, University of ZurichZürich, Switzerland; ^3^School of Biological Sciences, Institute for Global Food Security, Queen's University BelfastBelfast, UK

**Keywords:** bla genes, plasmid sequencing, CTX-M, TEM, accessory genes

## Abstract

**Objectives:** Previously 14 conjugative plasmids from multi-drug resistant (MDR) *Escherichia coli* from healthy humans and food-producing animals in Switzerland were sequenced. The aim of this study was to extend the genetic characterization of these plasmids with a focus on *bla*_ESBL_ genes including *bla*_CTX-M-1_ and *bla*_TEM_, class 1 integrons and toxin-antitoxin (TA) systems contained therein.

**Methods:** The nucleotide sequences and subsequent annotation therein of 14 conjugative plasmids were previously determined from their corresponding transconjugants. The TA loci were confirmed by RASTA-Bacteria.

**Results:** Eight of the conjugative plasmids identified were found to encode genes expressing ESBLs. Structural heterogeneity was noted in the regions flanking both the *bla*_CTX-M-1_ and *bla*_TEM_ genes. The *bla*_CTX-M-1_ genes were associated with the common insertion sequences IS*Ecp1* and IS*26*, and uniquely with an IS*5* element in one case; while *bla*_TEM_ genes were found to be associated with IS*26* and Tn*2*. A new *bla*_TEM-210_ gene was identified. Seven class 1 integrons were also identified and assigned into 3 groups, denoted as In54, In369 and In501. Sixteen TA loci belonging to 4 of the TA gene families (*relBE*, *vapBC*, *ccd* and *mazEF*) were identified on 11 of these plasmids.

**Conclusions:** Comparative sequence analysis of these plasmids provided data on the structures likely to contribute to sequence diversity associated with these accessory genes, including IS*26*, IS*Ecp1* and Tn*2*. All of them contribute to the dissemination of the corresponding resistance genes located on the different plasmids. There appears to be no association between β-lactam encoding genes and TA systems.

## Introduction

Understanding the molecular epidemiology of antimicrobial drug resistance genes has been a complex task due to the plasmids' role in the spread of these elements (Carattoli et al., 2006; Hopkins et al., 2006). Moreover, the diversity and promiscuity of resistance genes has become a major global public health issue since antimicrobial resistance threatens the effective prevention and treatment of an ever-increasing number of infections in a clinical setting. Plasmid-located genes can be acquired from several sources and disseminated by horizontal gene transfer (HGT). The latter strategy contributes to the dissemination of many of the undesirable phenotypes associated with bacteria, including antibiotic resistance, virulence, and resistance to heavy metals (Davies and Davies, 2010; Baquero et al., 2013). Transmissible plasmids can be considered as particularly successful entities within the communal gene pool, and those that encode a full set of conjugation-encoding genes are referred to as conjugative, facilitating their dissemination over large taxonomic distances (Norman et al., 2009). Of note, there is a need to standardize surveillance methods with an emphasis on gene tracking by plasmid sequencing as an aid to reveal the transmission of resistance among bacteria from animals to humans *vice versa*.

*Escherichia coli* producing extended-spectrum β-lactamases (ESBLs) is a major problem in worldwide, since these ESBL genes can be spread by plasmid-mediated integrons, insertion sequence (IS) elements, and transposons among different bacteria species causing outbreaks as well as sporadic infections (Liebana et al., 2013). Plasmids expressing an ESBL phenotype frequently carry genes encoding resistance to other commonly used antimicrobial drug classes and genes for toxin-antitoxin (TA) systems, which contribute to the maintenance of plasmids in their host. Thus, understanding the roles of these genetic elements and the surrounding genetic structures are essential and relevant to the subsequent development of strategies to limit the dissemination and persistence of ESBL genes and others.

In a recent publication (Wang et al., 2014), we reported the complete nucleotide sequences of 14 large (>30 kb) conjugative plasmids identified in nine multi-drug resistant (MDR) *E. coli* isolates expressing an ESBL phenotype isolated from food-producing animals and healthy humans in Switzerland. A comparative analysis of these plasmid-backbone structures was carried out previously (Wang et al., 2014). The aim of the present study was to analyse, in detail, the accessory genes from these plasmids for (i) the genetic context in which *bla*_CTX−M−1_ and *bla*_TEM_ genes were located, (ii) the structure of class 1 integrons and their gene cassettes, (iii) the identification of other related antimicrobial resistance genes and (iv) the identification of the TA systems.

## Materials and methods

### Bacterial isolates, susceptibility testing and genetic annotation

Nine ESBL-producing *E. coli* isolated from fecal samples of food-producing animals and healthy humans in Switzerland from 2009 to 2011 (Geser et al., 2012a,b), were identified previously. All of the associated technical protocols were described in earlier publications (Wang et al., 2013, 2014). The antimicrobial resistance profiles for these isolates are shown in a supplementary table (Table S1).

### Bioinformatics search for TA pairs

Putative TA systems were identified in the 14 conjugative plasmids using the RASTA-Bacteria program version 2.12 (http://genoweb1.irisa.fr/duals/RASTA-Bacteria/). A summary of the TA features associated with the plasmids is shown in Table 1.

**Table 1:** **Summary of the TA features associated with all 14 conjugative sequenced plasmids from healthy human and food-producing animal origin previously reported in Switzerland**.

**Plasmid name**	**Accession no**.	**Plasmid size (bp)**	**Inc-type/pMLST**	**IS**	**Integron/Transposon**	**β-Lactamase(s) identified**	**Toxin-Antitoxin genes**
pH2332-166	KJ484626	166,594	IncFII-IncFIB	**–**	In369	TEM-1	*vapC/vagC; relE/relB*
pH2332-107	KJ484627	107,386	IncB/O	IS*26*-ΔIS*Ecp1*; IS*26*	**–**	CTX-M-1	*relE/relB*
pH2291-144	KJ484628	144,925	IncFII-IncFIB	**–**	In369; Tn*2*	TEM-1	*vapC/vagC*
pH2291-112	KJ484629	112,671	IncI1/ST3	IS*Ecp1*; IS*5*	In54	CTX-M-1	*relE/relB*
pH1519-88	KJ484630	88,678	IncI1/ST145	IS*Ecp1*	Tn*2*	TEM-210; CTX-M-1	none identified
pH1519-76	KJ484631	76,197	IncFII	**–**	**–**	**–**	none identified
pH1038-142	KJ484634	142,875	IncF-IncN/ST1	IS*26*-ΔIS*Ecp1*; IS*26*	In501	TEM-1; CTX-M-1	*mazF/maze*
pC60-108	KJ484635	108,662	IncI1/ST3	IS*Ecp1*	In54	CTX-M-1	*relE/relB*
pC59-153	KJ484636	153,231	IncFIIA-IncFIC-IncFIB	**–**	**–**	**–**	*vapC-1/vagC-1; vapC-2/vagC-2; vapC-3/vagC-3; ccdB/ccdA; relE/relB*
pC59-112	KJ484637	112,330	IncI1/ST3	IS*Ecp1*	In54	CTX-M-1	*relE/relB*
pC49-108	KJ484638	108,660	IncI1/ST3	IS*Ecp1*	In54	CTX-M-1	*relE/relB*
pC23-89	KJ484639	89,513	IncI1/ST36	**–**	Tn*2*	TEM-52	*vapC/vagC*
pL2-87	KJ484640	87,042	IncB/O	**–**	**–**	**–**	*relE/relB*
pL2-43	KJ484641	43,265	IncN/ST1	IS*26*-ΔIS*Ecp1*; IS*26*	**–**	CTX-M-1	none identified

*–Feature not identified*.

### Reference numbers for class 1 integrons

Standard methods were used to annotate the class 1 integrons by INTEGRALL platform version 1.2 (http://integrall.bio.ua.pt/), referred as In501, In369 or In54 (**Figure 3**).

## Results and discussion

### Comparison of the genetic environment of bla_CTX−M−1_ genes

**Figure 1:**
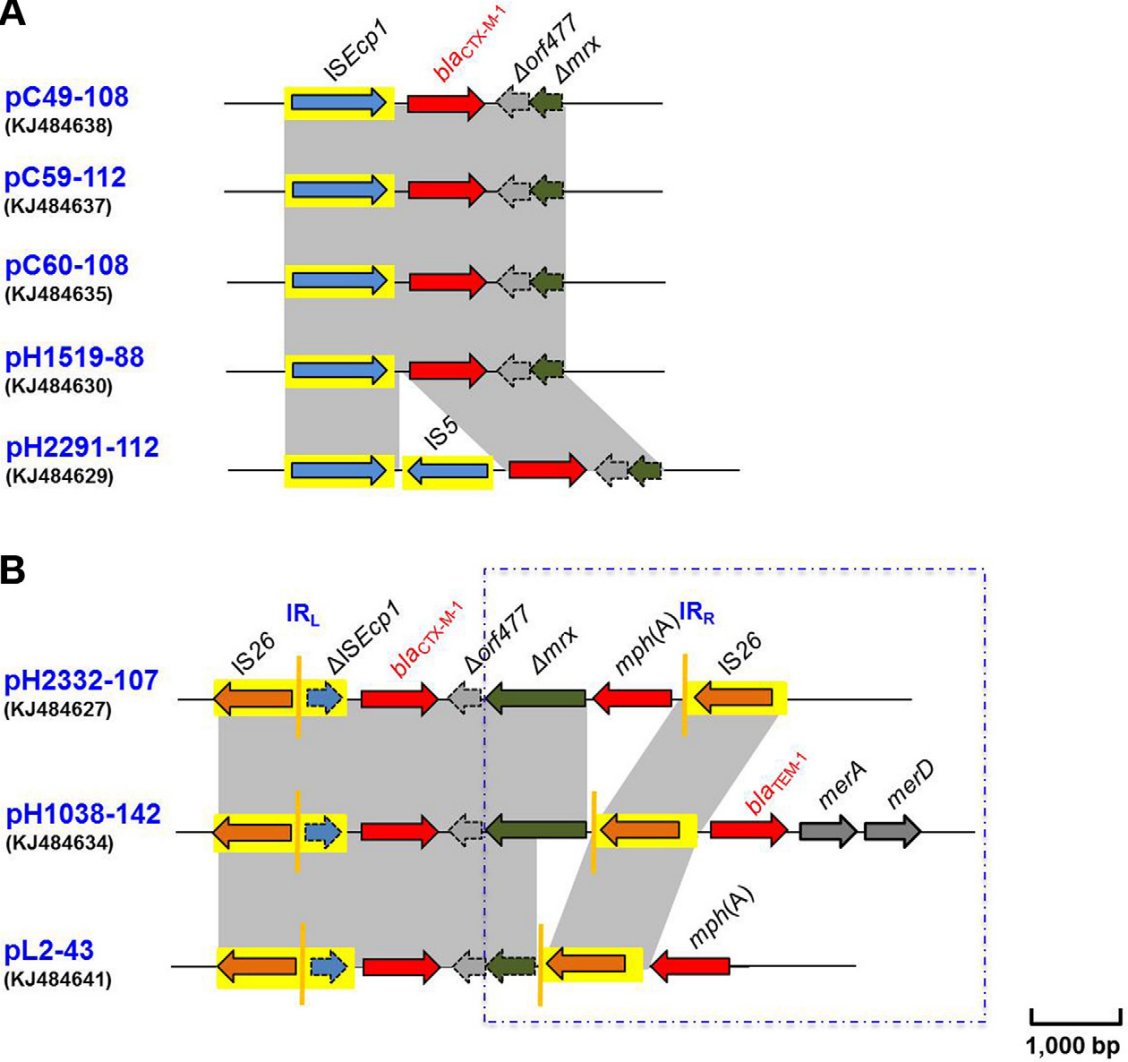
**(A)** Comparative schematic representation of the flanking regions of the *bla*_CTX-M-1_ genes in plasmids pC49-108, pC59-112, pC60-108, pH1519-88, and pH2291-112. Areas shades in gray indicate homologies in the corresponding genetic environment on each plasmid. The ORFs are shown as arrows, with the arrowhead indicating the direction of transcription. IS*Ecp1* elements are shown as arrowed boxes. **(B)** Comparative schematic representation of the flanking regions of the *bla*_CTX-M-1_ genes in plasmids pH2332-107, pH1038-142, and pL2-43. Areas shades in gray indicate homologies in the corresponding genetic environment on each plasmid. The ORFs are shown as arrows, with the arrowhead indicating the direction of transcription. IS elements are shown as arrowed boxes.

Eight *bla*_CTX−M−1_ genes were found to be located within various genetic contexts in the present study. Either a complete or partial IS*Ecp1* gene was identified proximal to the resistance gene (Figure 1). A complete IS*Ecp1* gene was more often identified (Figure 1A), along with a partial ORF (denoted as Δ*orf477*) and a Δ*mrx* gene located distally in this arrangement. In three of the eight loci studied, a novel genetic arrangement was also noted wherein a truncated copy of IS*Ecp1* gene was identified (denoted as ΔIS*Ecp1*) and which proximally flanked the *bla*_CTX−M−1_ genes with an IS*26* element being located further upstream (Figure 1B).

Further consideration of the eight *bla*_CTX−M−1_ containing plasmids suggested that they could be divided into two groups based on their different disrupted insertion sequence elements. The first group consisted of five of the eight plasmids, including pC49-108, pC59-112, pC60-108, pH1519-88, and pH2291-112 (Figure 1A and Table 1). All 5 plasmids were typed as IncI1 and showed a high degree of similarity (99% at the nucleotide level) with the reference IncI1 plasmid R64 (Table 1) (Wang et al., 2014). With the exception of pH2291-112, a *tnpA* gene of 1263 bp was found to be located upstream of the four *bla*_CTX−M−1_ genes (Figure 1A). This was typical of the genetic organization previously identified in *bla*_CTX−M−1_ containing plasmids, and the former DNA sequence was determined to be 100% identical to IS*Ecp1* (accession no.: AJ242809) in all cases. IS*Ecp1* was originally identified proximal to the plasmid-mediated cephalosporinase gene *bla*_CMY−4_ in an *E. coli* isolate identified in the United Kingdom (accession no.: AJ242809). This unusual IS element was also commonly found to be associated with several *bla*_CTX−M_ genes (Figure S1) (Karim et al., 2001; Saladin et al., 2002; Poirel et al., 2003; Lartigue et al., 2004). IS*Ecp1* elements are known to play a dual role, acting both as a transposase and ensuring the expression of the downstream ORF *via* a strong putative promoter (Poirel et al., 2003, 2005; Hossain et al., 2004; Jacoby, 2009). In the case of pH2291-112, unusually the locus between the latter transposon and the β-lactamase resistance gene was interrupted by an IS*5* element (Figure 1A).

Analysis of the DNA downstream of the *bla*_CTX−M−1_ genes showed a varied gene organization. In the CTX-M-1 cluster, a partial sequence of 342 bp denoted as Δ*orf477* was present in all structures studied and followed by a partially deleted Δ*mrx* gene of 110 bp. This organization (IS*Ecp1*- *bla*_CTX−M−1_-Δ*orf477*-Δ*mrx*) was determined to be identical to the corresponding region found in other IncI1 plasmid pCTX1261 (accession no.: HF549089) and also in an IncF plasmid pCTX2412 (accession no.: HF549091) originally purified from *E. coli* cultured from swine and horse samples in Germany (Schink et al., 2013). The data reported here further confirmed the importance of IS*Ecp1* that is thought to function in the mobilization of *bla*_CTX−M_ genes among IncI1 type plasmids.

The insertion sequence element IS*5* identified upstream of the *bla*_CTX−M−1_ gene in pH2291-112 (Figure 1A) has only been reported on one other plasmid previously. The IS*Ecp1*-IS*5* arrangement was originally identified on a *Klebsiella pneumoniae* plasmid p9701 (accession no.: FM246881) and this was associated with the mobilization of *bla*_CMY−2_ gene (Figure S1B) (Verdet et al., 2009). Moreover, this arrangement was recently reported in the genomic DNA of *E. coli* EC958, which was associated with one *bla*_CMY−23_ gene (Figure S1B) (Totsika et al., 2011).

The second group was unique in that a truncated copy of the IS*Ecp1* transposon (denoted as ΔIS*Ecp1*) was identified and found to be located between the *bla*_CTX−M−1_ and an IS*26*. This unusual arrangement was identified in the remaining three plasmids: pH2332-107 (IncB/O), pH1038-142 (IncF-IncN), and pL2-43 (IncN) (Figure 1B). In all cases, the truncated ΔIS*Ecp1* was 214 bp in length. All of these sequences contained the putative promoter region involved in the transcription of *bla*_CTX−M_ genes. When the region proximal to the *bla*_CTX−M−1_ gene was considered, it showed some unusual features. In the case of pH2332-107, the distal region of this gene consisted of the terminal 342 bp of *orf477*, followed by a partially deleted *mrx* gene (Δ*mrx*), an entire *mph*(A) gene and a second copy of IS*26* (Figure 1B). The gene module IS*26*-ΔIS*Ecp1*-*bla*_CTX−M−1_−Δ*mrx*-*mph*(A)-IS*26* was recently identified in plasmids purified from *E. coli*, including those from the successful clone ST131 and from clinical *E. coli* isolated from pigs in Germany (Cullik et al., 2010). In particular, this structure had been identified in several IncN and IncI1 plasmids (Schink et al., 2011; Dolejska et al., 2013). Since the identical structure of the *bla*_CTX−M−1_ region can be found in plasmids of different incompatibility groups, it has been hypothesized that the complete structure can be exchanged *en-bloc* between different plasmid backbones as a composite transposon mediated by an IS*26* transposition event (Cullik et al., 2010; Dolejska et al., 2013).

In contrast to the genetic arrangement above, in pH1038-142, the locus containing the *mph*(A) gene was deleted and extended beyond the distal IS*26* with a region containing another gene, *bla*_TEM−1_ in addition to the *merA* and *merD* genes, encoding resistance to mercury (Figure 1B). Based on our extensive searching of the current databases, this is the first description of such an arrangement. Further examination of the *bla*_CTX−M−1_ region in pL2-43 revealed other novel structural rearrangements. As shown in Figure 1B, similar to the situation described above for pH1038-142, the distal IS*26* element, with intact left and right inverted repeats, was further extended with a copy of *mph*(A) followed by a hypothetical ORF (not shown in Figure 1B). These findings suggest that perhaps IS*26* may play a hitherto unrecognized but important role in the mobilization of these ESBL genes.

### Comparison of the genetic environment of bla_TEM_ regions

**Figure 2:**
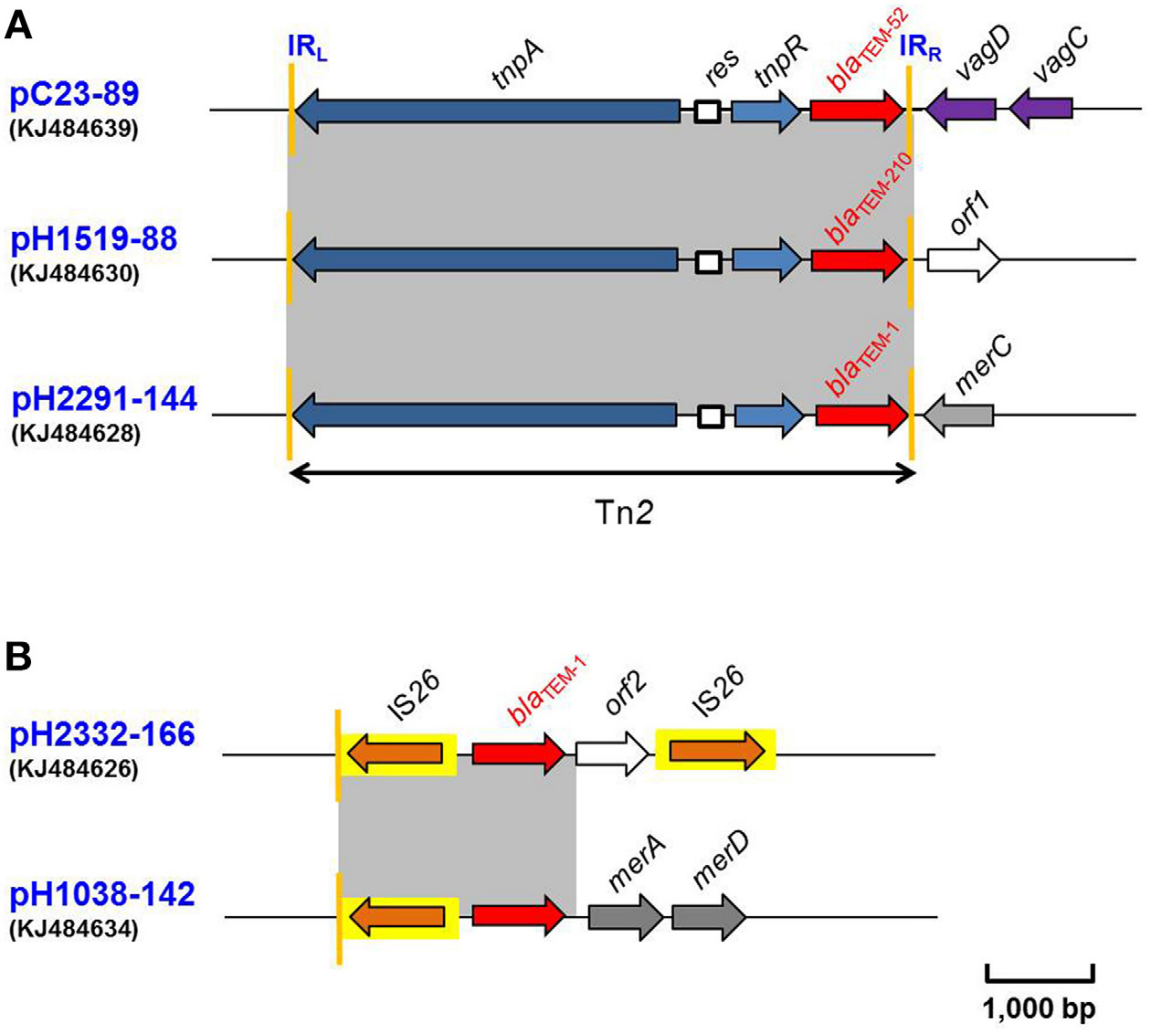
**(A,B)** Schematic presentation of the flanking regions of the *bla*_TEM_ genes in plasmids pC23-89, pH1519-88, pH2291-144, pH2332-166, and pH1038-142. Areas shades in gray indicate homologies in the corresponding genetic region on each plasmid. The ORFs are shown as arrows, with the arrowhead indicating the direction of transcription. IS elements are shown as arrowed boxes.

Five *bla*_TEM_ genes were identified in this study. These were found to be located within three different genetic environments (Figure 2). In two of these (pH1519-88 and pH1038-142) the *bla*_TEM_ gene was located on the same plasmid containing a *bla*_CTX−M−1_ gene. A complex transposon Tn*2* was identified on three of these plasmids, pC23-89 (IncI1) containing *bla*_TEM−52_, pH1519-88 (IncI1) containing a new variant *bla*_TEM−210_ and pH2291-144 (IncFII-IncFIB) containing *bla*_TEM−1_ (Figure 2A). In this case, the β-lactamase-encoding resistance gene was located distal to the *tnpR*-encoding gene. In the remaining two cases, the *bla*_TEM−1_ gene was flanked by two copies of an IS*26* element as shown for plasmid pH2332-166 (IncFII-IncFIB) and a proximally located IS*26* element in pH1038-142 (IncF-IncN), followed by *merA*-*merD* genes (Figure 2B).

The single *bla*_TEM−52_-carrying plasmid, pC23-89 was purified from an *E. coli* isolate of poultry origin and belonged to replicon type IncI1. Sequence analysis of the immediate flanking regions identified *tnpA* and *tnpR* genes of transposon Tn*2* located upstream of the *bla*_TEM−52_ gene. The right inverted repeat (denoted as IR_R_ in Figure 2A) of Tn*2* was followed by the virulence-associated *vagD* and *vagC* genes (Figure 2A). These alleles showed 100% nucleotide identity with another two IncI1 plasmids, which also harbored *bla*_TEM−52_ genes. One (accession no.: EF141186) was identified in a *Salmonella* Infantis, isolated in Belgium in 2005 (Cloeckaert et al., 2007); and the other was located on plasmid pESBL-117 (accession no.: CP008734) from an ESBL-producing *E. coli* isolate recovered in Netherlands in 2014 (Brouwer et al., 2014). Recently, this *bla*_TEM−52_ gene had been reported within a partially-sequenced Tn2 transposon derivative on an IncI1 plasmid, pCTX909 (accession no.: HF549095) from a poultry source in Germany (Schink et al., 2013). [Bailey and coworkers (Bailey et al., 2011) reported that transposon Tn*2* is annotated incorrectly as Tn*3* transposon in the respective database entry].

Highly transmissible IncI1 plasmids carrying the *bla*_TEM−52_ are of particular interest as they are globally spread among *E. coli* populations from humans and animals (Carattoli, 2009). IncI1 plasmids carrying *bla*_TEM−52_ within transposon Tn*2*, similar to that observed for pC23-89 (Figure 2A), have been described in *Salmonella enterica* and *E. coli* isolates from poultry and humans in Belgium, Germany and the Netherlands, respectively, (Cloeckaert et al., 2007; Leverstein-Van Hall et al., 2011) and these appear to be widely distributed among different members of the Enterobacteriaceae family. Furthermore, a range of additional TEM variants (including TEM-20, −52, and −126) have similarly been detected in food-producing animals and from isolates cultured from foods in different European countries (De Champs et al., 2004; Sunde et al., 2009). Moreover, in relation to the ESBLs of the TEM class, the most frequently detected throughout the EU was reported to be TEM-52 [EFSA Panel on Biological Hazards (BIOHAZ), 2011].

Based on the sequencing data from pH1519-88, a novel TEM variant was identified and classified as *bla*_TEM−210_ (Figure 2A). Plasmid pH1519-88 also belonged to the IncI1 group. Sequence analysis of *bla*_TEM−210_ revealed two mutations which gave rise to two amino acid substitutions at positions corresponding to residues 49 and 69 when compared with a previously published sequence (accession no.: AF309824). The *bla*_TEM−210_ gene had also been identified within a Tn*2* transposon (Figure 2A).

The Tn*2*-*bla*_TEM−1_ region of 4770 bp in length located on pH2291-144 exhibited 100% DNA sequence identity to that found in several plasmids in *E. coli*, including the conjugative plasmid pVQS1 (accession no.: JQ609357). The latter carries a *qnrS1* gene associated with an IncN group and was originally identified in a *Salmonella* Virchow isolate, which was cultured from a patient in Switzerland returning from a foreign holiday (Karczmarczyk et al., 2012). It is interesting to note that, whereas pH2291-144 and pH2332-166 showed 99% nucleotide identity across their backbones, the *bla*_TEM−1_ genes contained on these plasmids were associated with different transposons (Figures 2A,B). The *bla*_TEM−1_ gene of pH2291-144 was located within Tn*2* and inserted within a *mer* module (Figure 2A), whereas in pH2332-166, the *bla*_TEM−1_ was flanked by two copies of IS*26* (Figure 2B).

Several *bla*_TEM_ genes have been reported to be associated with different transposons, and with Tn*2* in particular as the dominant *bla*_TEM_-containing transposon in commensal *E. coli* (Bailey et al., 2011). Although information on the genetic context of the *bla*_TEM_ genes would be valuable in extending our understanding of how this important resistance gene is disseminated, for most TEM variants little or no sequence beyond the immediate flanking regions of the gene is available (Bailey et al., 2011).

### Comparison of class 1 integron regions

**Figure 3:**
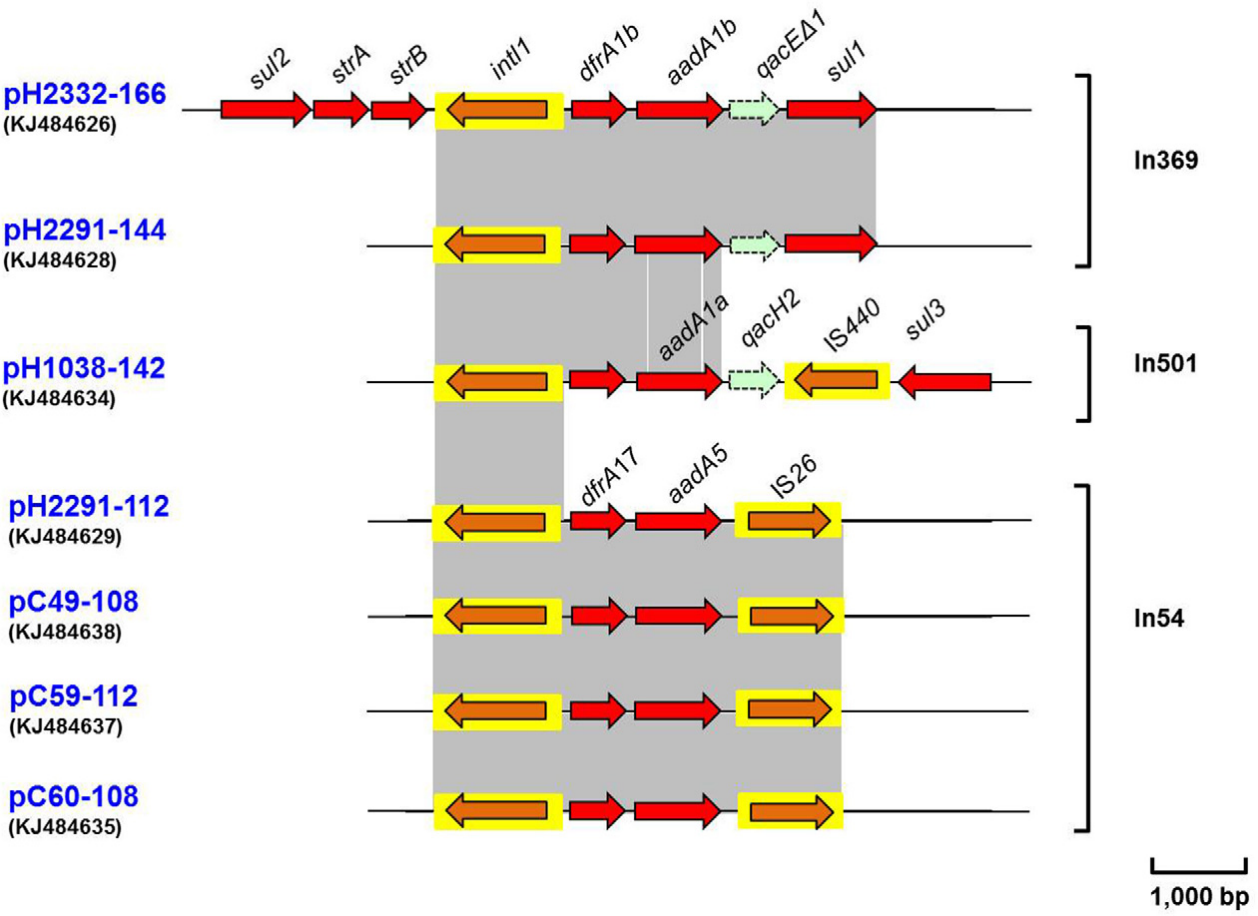
**Schematic presentation showing the complete class I integrin along with the flanking regions identified on plasmids pH2332-166, pH2291-144, pH1038-142, pH2291-112, pC49-108, pC59-112, and pC60-108**. Areas shaded in gray and the arrangements of the ORFs are as indicated in Figure 2 above. IS elements are shown as arrowed boxes.

Several complete class 1 integron structures were noted among the 14 sequenced conjugative plasmids. Typically a class 1 integron is composed of three key features; a 5′-conserved structure (CS) containing an *int1* site-specific recombinase; followed by a variable region containing one or more gene cassettes and then the 3′-CS containing the *qacEΔ1* and a *sul1*-encoding genes. A complete class 1 integron was identified in plasmids pH2332-166 and pH2291-144 (Figure 3), wherein two gene cassettes *dfrA1b-aadA1b* were identified in the typical *head-to-tail* arrangement. Furthermore, on pH2332-166, three antimicrobial resistance genes, *strB, strA* and *sul2*, that encode resistance to streptomycin and sulphonamide, were identified and found to flank the 5′-CS region of a complete class 1 integron; these latter genes were lacking on pH2291-144 (Figure 3). Sequence analysis of the segment (*sul2*-*strA*-*strB*-*intI1*-*dfrA1b*-*aadA1b*-*qacEΔ1*-*sul1*) (Figure 3) on pH2332-166 exhibited 100% similarity to the corresponding sequence located on the plasmid pNRG857c (accession no.: CP001856), which was previously isolated from a clinical isolate of adherent and invasive *E. coli* in Germany (Nash et al., 2010).

Located on pH1038-142 was the gene cassette containing *dfrA1b*-*aadA1a* and this was located distally to a 5′-CS which in this case was devoid of a complete 3′-CS, being replaced instead by a single copy of *qacH2* and an insertion sequence element, IS*440* (Figure 3). Downstream of the latter element was a *sul3* gene that encodes resistance to sulphonamides. The cluster of *intl1*-*dfrA1b*-*aadA1a*-*qacH2*-IS*440*-*sul3* shared 100% identity to the corresponding DNA sequence with plasmid pEC54 (accession no.: FM244709), which was isolated from one *sul3*-encoding *E. coli* isolate of porcine origin in Germany.

A similarly structured class 1 integron was also identified from the R64-like IncI1 plasmids, including pH2291-112, pC49-108, pC59-112 and pC60-108; wherein the 3′-CS was replaced by IS*26* (Figure 3). The gene cassette array of *intl1*-*dfrA17*-*aadA5*-IS*26* (3,470 bp) exhibited 100% nucleotide sequence identity to that of the class 1 integron in *Salmonella enteric* serovar Indiana located on a non-conjugative plasmid pS414 from Shandong, China, 2008 (accession no.: KC237285) (Lai et al., 2013).

All seven integron sequences identified in this study were deposited in the INTEGRALL database (http://integrall.bio.ua.pt/) and were designated as In369, In54 or In501 (Figure 3).

### Other related antimicrobial resistance genes

**Figure 4:**
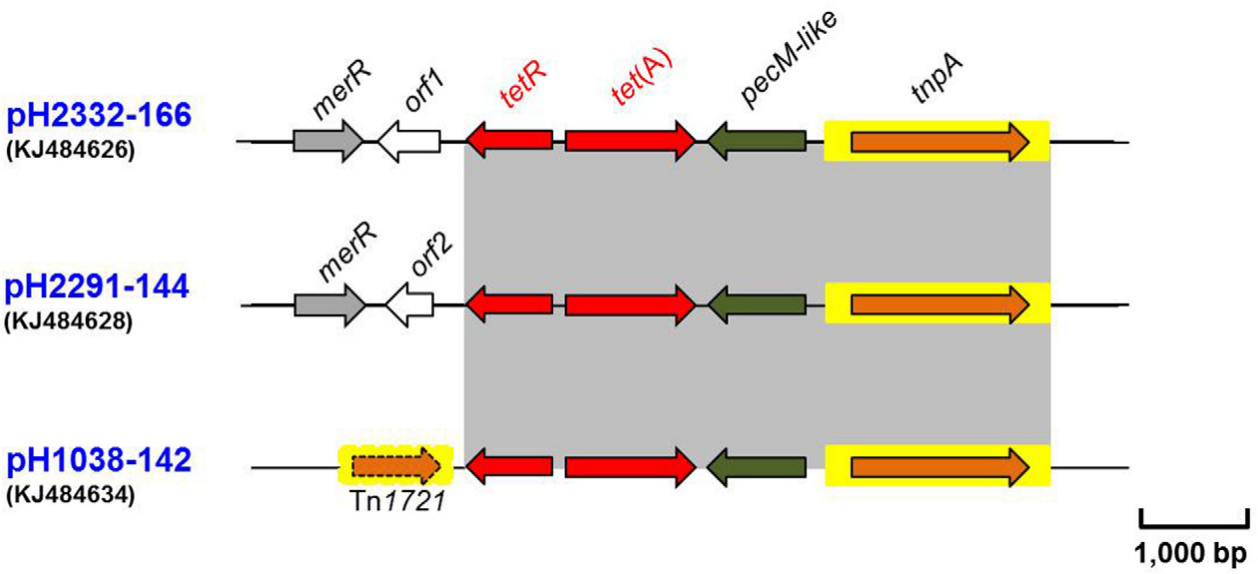
**Schematic presentation of the flanking gene regions of the *tetAR*-modules identified on plasmids pH2332-166, pH2291-144, and pH1038-142**. Areas shaded in gray and the arrangements of the ORFs are as indicated in Figure 2 above. IS elements are shown as arrowed boxes.

Three *tetAR* modules coding for a class A tetracycline efflux protein and a repressor, were identified on pH2332-166, pH2291-144 and pH1038-142 respectively (Figure 4 and Table 1). The loci (*tnpA*-*pecM*-*tet*(A)-*tetR*) showed a high degree of sequence similarity to each other, at 99% nucleotide identity. The *tet*(A) gene on each plasmid was flanked on the distal side by a *pecM* gene encoding a PecM-like protein, which has been reported previously to be associated with *tetAR* genes (Pasquali et al., 2005; Szczepanowski et al., 2011). On pH2332-166 and pH2291-144, the *tetR* genes were located on the distal side of a mercury resistance cassette (Wang et al., 2014). In plasmid pH1038-142 this module was located between a class 1 integron and a *strAB* module. It has been suggested previously that the *tetAR* and adjacent genes serve as a *hot-spot* for insertion of different mobile elements and can be modified by direct-repeat-mediated deletional events (Szczepanowski et al., 2011). It is conceivable that additional resistance modules could be integrated into the *tet*-platform.

A *sul2*-*strA*-*strB* gene cluster was noted on pH2332-166 (Figure 3), encoding resistance to sulphonamide and streptomycin, which showed a high degree of similarity with that of plasmid pCERC1 (accession no.: JN012467), at 99% nucleotide identity. The plasmid pCERC1 carrying the *dfrA14* cassette in the *strA* gene of the *sul2*-*strA*-*strB* gene cluster, has been reported in many countries, indicating a global distribution and it appears to have been circulating in Gram-negative bacteria for more than 25 years (Anantham and Hall, 2012).

All of the transconjugant strains were re-screened for antimicrobial resistance genes as previously described (Karczmarczyk et al., 2011). The antimicrobial resistance profiles for the nine strains in this study are shown in a supplementary table (Table S1).

### TA systems identified on plasmids isolated from *E. coli* in healthy food-producing animals and humans

TA loci play a role in bacterial stress physiology and the stabilization of horizontally acquired elements. TA systems are composed of a toxin-encoding gene along with its cognate antitoxin and these features prevent *post-segregational killing* by the host bacterial cell. In addition, these genetic loci also act to eliminate compatible plasmids, therefore ensuring the maintenance of the plasmid in the bacterial cell during replication (Hayes, 2003; Unterholzner et al., 2013). In adapted *E. coli*, it has been proposed, previously that strain virulence and the association with IncF plasmids, could contribute to the success and spread of the *bla*_CTX−M−15_ genes (Clermont et al., 2008; Mnif et al., 2010). Furthermore, recent experiments reported that ESBL-plasmids carrying TA systems can be *cured* from field isolates of *E. coli* using a heat technique, and the *cured* ESBL-plasmids contained at least one complete TA system, whose loss would normally mean the death of bacterial cells (Schaufler et al., 2013). TA loci-encoded toxins themselves may represent a novel target that could be exploited for the development of future generation of antibacterial compounds (Gupta, 2009).

Using BLASTP and RASTA-Bacteria, we searched all 14 conjugative plasmids and identified four different TA families (Table S2). Six TA loci, denoted as VapC (toxin protein) and VagC (antitoxin/virulence-associated protein), were identified on plasmids pH2332-166, pH2291-144, pC59-153, and pC23-89. The addiction module toxin RelE and antitoxin RelB protein were detected from eight plasmids, pH2332-166, pH2332-107, pH2291-112, pC60-108, pC59-153, pC59-112, pC49-108, and pL2-87, respectively. A representative of the *mazEF* family was located on the multidrug resistant plasmid pH1038-142. The plasmid maintenance protein, toxin CcdB and antitoxin CcdA protein, were only detected on plasmid pC59-153. A summary of TA systems from the 14 conjugative plasmids and the corresponding protein sequences are shown in Table S2.

**Figure 5:**
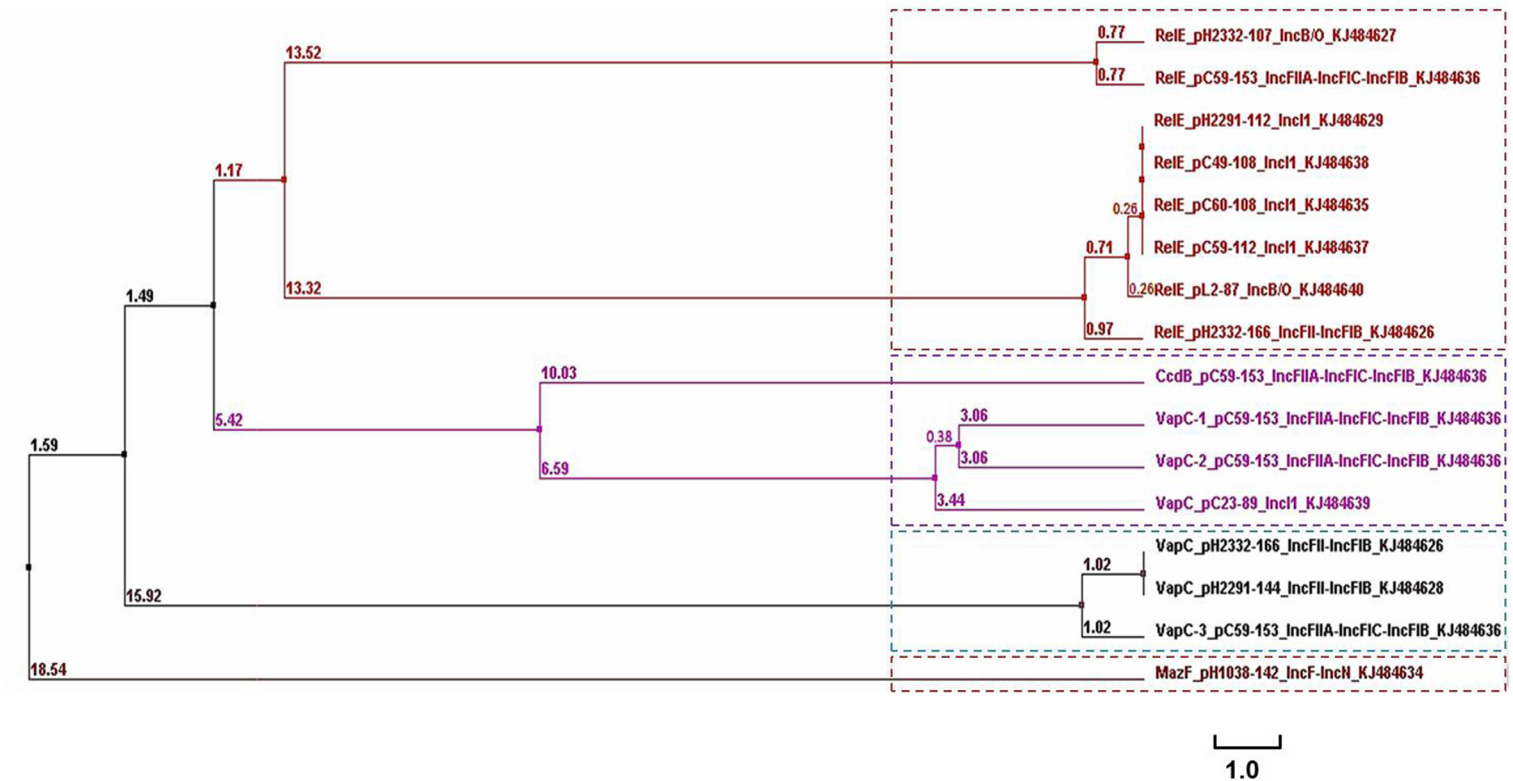
**Phylogenetic tree (chladogram) for the amino acid sequences of plasmid-encoded toxin genes from 14 conjugative plasmids (see Table 1)**. The TA loci sequences were retrieved from Table S2 (raw sequences). The tree was calculated using Clustal Omega version 1.2.1. The lengths of the horizontal lines indicate relative evolutionary distances. A scale bar is also shown.

A phylogenetic tree containing the RelE/VapC/CcdB/MazF superfamily toxins identified on these plasmids in this study is shown in Figure 5. Four clusters containing these toxin sequences were noted. The RelE superfamily was located on plasmids with different Inc types in this study (Figure 5 and Table 1). In IncI1 plasmids, the RelE sequences were identical, with variations being evident from the clustering when located in plasmids of IncB/O or IncF types (Figure 5). It is also interesting to note that pC59-153 possessed three *vapC* toxin-encoding genes, which were located in different plasmid genomes. Additionally, the backbone of pC59-153 showed high levels of nucleotide similarity with several ColV plasmids that are known to be associated with an avian pathogenic *E. coli* strains (Wang et al., 2014). Thus, the presence of multiple *vapC* toxin-encoding genes and other TA systems in pC59-153 could support plasmid stability and virulence. Figure 5 shows that the six VapC sequences cluster into two different groups according to their amino acid sequence similarities, and all but one belonged to IncF types. While the relationship between the TA systems and the plasmid incompatibility types remains unclear, further study may help in the understanding of the nature of these TA systems in plasmids and their specific physiological role(s) in bacteria. Amino acid sequence similarity and clustering analysis of the antitoxin genes were carried out and these showed similar characteristics to their corresponding toxin genes (data not shown).

In many cases, TA loci were clustered or closely linked to mobile genetic elements (Pandey and Gerdes, 2005). In the most extreme of these cases, three *vapC/vagC* loci from plasmids pH2332-166, pH2291-144, and pC59-153, were found to be located in the *traD*-*traI* intergenic region. All *relBE* homologous loci identified in our study, were *bona fide* integron elements located between replication proteins and mobile element proteins. It is tempting to speculate that these features suggest that TA loci may represent a type of mobile cassette that can move between plasmids of different Inc types or different original bacterial sources and that undergo rapid evolution and horizontal transfer.

### Concluding remarks

In this study we extended our careful analysis of the accessory genes in 14 conjugative plasmids from nine unrelated human, poultry and lamb *E. coli* isolates. These plasmids have the capacity to transfer at high frequency to suitable recipient bacterial strains, providing a means by which the *bla*_ESBL_ genes harbored therein can be quickly disseminated. This feature may in turn compromise the use of this class of antibiotic for the treatment of infections in both animals and humans alike. Furthermore it suggests the existence of a reservoir of resistance genes that is highly mobile and contained in more than one genetic context. Our current study reports on some novel arrangements that were identified in the plasmids conferring resistance to β-lactam and other commonly-used antibiotics, and the structural heterogeneity associated with these regions flanking antibiotic resistance genes. Insertion sequences and transposons, such as IS*Ecp1*, IS*26*, and Tn*2*, are likely to contribute to the dissemination of the *bla*_CTX−M−1_ and *bla*_TEM_ gene containing regions between different ecosystems (human and animal). Seven class 1 integrons were identified and designated into three classes; and 16 TA loci from 11 of these plasmids were assigned into four groups according to their toxin sequence similarity. There appeared to be no link between the β-lactam resistance-encoding genes and the TA systems in this study. Moreover, the nature of specific addiction systems appears to differ with the type of replicon with no obvious epidemiological links. Nonetheless, these antimicrobial resistance genes and addiction modules are located on efficient conjugative plasmids (i.e., IncI1 and IncF-related), and this could contribute to the emergence and spread of these genes, in different bacterial hosts. Taken together these characteristics provide much of the motivation for extending our understanding of the molecular epidemiology and structural relationships of accessory genes that exist between plasmids from a variety of Inc types and bacterial sources. Further studies will be necessary to understand the driving forces associated with accessory gene cassettes transfer and plasmid spreading.

## Funding

This study was supported by internal funding.

### Conflict of interest statement

The authors declare that the research was conducted in the absence of any commercial or financial relationships that could be construed as a potential conflict of interest.

## Supplementary material

Figure S1 **(A)** Comparison of the *bla*_CTX−M−1_ gene environment in plasmid pC49-108 with the *bla*_CTX−M−15_ gene in plasmid pEC_B24, and the *bla*_CMY−6_ gene in plasmid pA/C-LS6-SC29. Areas shades in gray indicate homologies in the corresponding genetic region on each plasmid. The ORFs are shown as arrows, with the arrowhead indicating the direction of transcription. IS*Ecp1* elements are shown as boxes with an arrow indicating the transcription orientation. **(B)** Comparison of the *bla*_CTX−M−1_ gene environment in plasmid pH2291-112 with the *bla*_CMY−2_ gene in plasmid p9701. Areas shades in gray indicate homologies in the corresponding genetic region on each plasmid. The ORFs are shown as arrows, with the arrowhead indicating the direction of transcription. IS*Ecp1* and IS*5* elements are shown as boxes with an arrow indicating their transcription orientation.
