# Supplementary figures and images for: Characterization of the genetic environment of blaESBL genes, integrons and toxin-antitoxin systems identified on large transferrable plasmids in multi-drug resistant Escherichia coli
Source: Front Microbiol. 2015 Jan 6;5:716. doi: 10.3389/fmicb.2014.00716 (PMC4285173; doi:10.3389/fmicb.2014.00716)

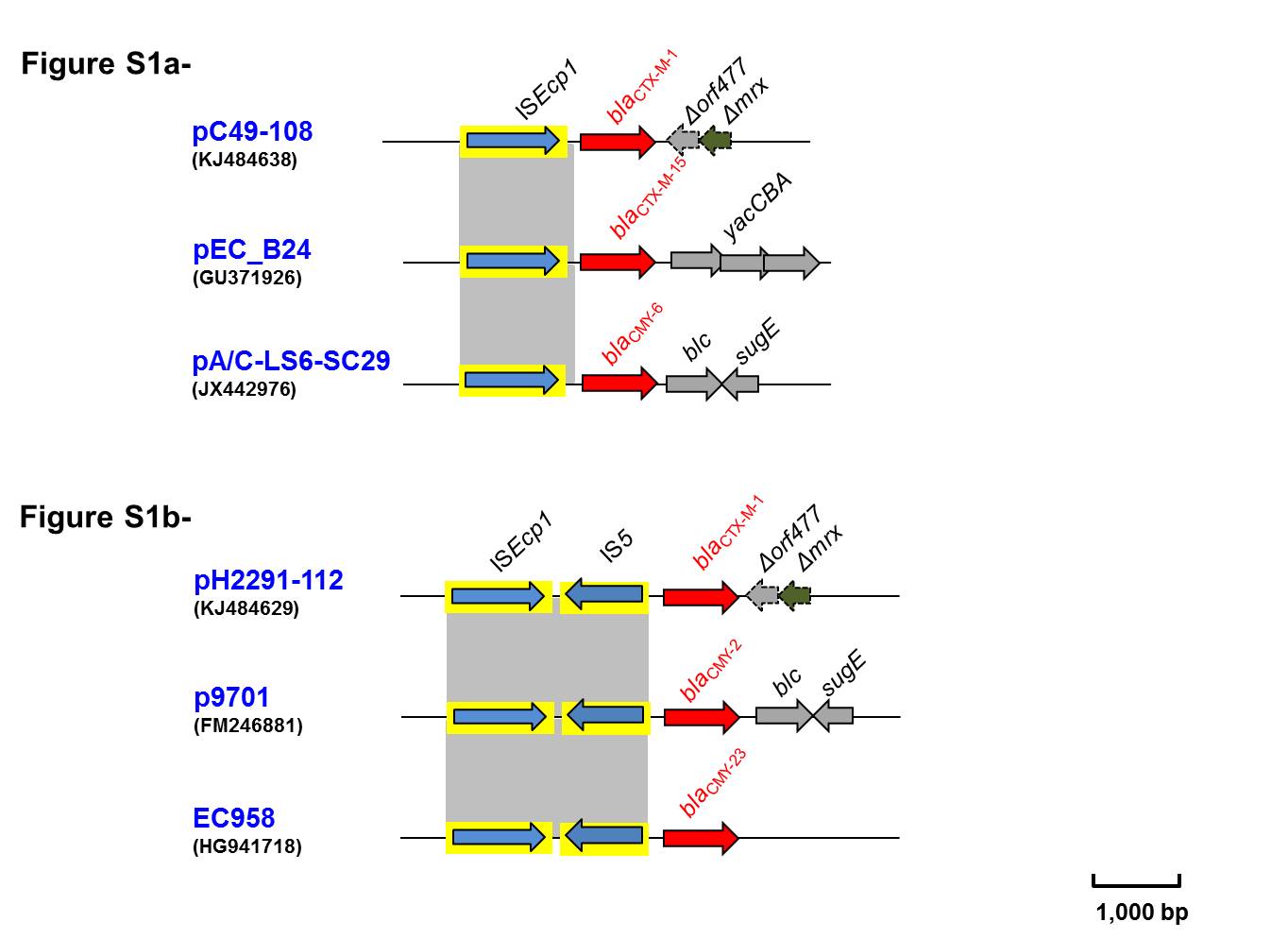

Supplement: Supplementary file 1 [file Image1.JPEG]
