# Supplementary material for: Characterization of the genetic environment of blaESBL genes, integrons and toxin-antitoxin systems identified on large transferrable plasmids in multi-drug resistant Escherichia coli
Source: Front Microbiol. 2015 Jan 6;5:716. doi: 10.3389/fmicb.2014.00716 (PMC4285173; doi:10.3389/fmicb.2014.00716)
Supplement: Supplementary file 2 [file Table1.DOCX]

**Supplementary Table S1-** Antimicrobial susceptibility of original bacterial isolates and their corresponding transconjugants (T) in this study

| **Isolate** | **Origin** |  | **Antimicrobial resistant phenotype** | | | | | | | | | |
| --- | --- | --- | --- | --- | --- | --- | --- | --- | --- | --- | --- | --- |
|  |  | **AMP** | **AMC** | **CHL** | **CTX** | **KAN** | | **CEP** | **STR** | **SXT** | **TET** | **TMP** |
|  |  |  |  |  |  |  |  |  |  |  |  |  |
| H-2332 | Human | **r** | s | **r** | **r** | s | | **r** | **r** | **r** | **r** | **r** |
| H-2332-(T) | - | **r** | s | **r** | **r** | s | | **r** | **r** | **r** | **r** | **r** |
| H-2291 | Human | **r** | s | s | **r** | s | | **r** | i | **r** | **r** | **r** |
| H-2291-(T) | - | **r** | s | s | **r** | s | | **r** | i | **r** | **r** | **r** |
| H-1519 | Human | **r** | **r** | s | **r** | s | | **r** | s | s | s | s |
| H-1519-(T) | - | **r** | **r** | s | **r** | s | | **r** | s | s | s | s |
| H-1038 | Human | **r** | s | **r** | **r** | s | | **r** | **r** | **r** | **r** | **r** |
| H-1038-(T) | - | **r** | s | **r** | **r** | s | | **r** | **r** | **r** | **r** | **r** |
| C-60 | Chicken | **r** | s | s | **r** | s | | **r** | i | **r** | **r** | **r** |
| C-60-(T) | - | **r** | s | s | **r** | s | | **r** | i | **r** | s | **r** |
| C-59 | Chicken | **r** | s | **r** | **r** | s | | **r** | i | **r** | s | **r** |
| C-59-(T) | - | **r** | s | **r** | **r** | s | | **r** | i | **r** | s | **r** |
| C-49 | Chicken | **r** | s | s | **r** | s | | **r** | i | **r** | **r** | **r** |
| C-49-(T) | - | **r** | s | s | **r** | s | | **r** | i | **r** | s | **r** |
| C-23 | Chicken | **r** | s | s | **r** | s | | **r** | s | s | s | s |
| C-23-(T) | - | **r** | s | s | **r** | s | | **r** | s | s | s | s |
| L-2 | Lamb | **r** | s | **r** | **r** | s | | **r** | **r** | s | **r** | s |
| L-2-(T) | - | **r** | s | s | **r** | s | | **r** | s | s | s | s |

a) s, susceptible; i, intermediate; r, resistant, based on CLSI guidelines.

b) Abbreviations: AMP, ampicillin, 10 μg; AMC, amoxicillin/clavulanic acid, 20/10 μg; CHL, chloramphenicol, 30 μg; CTX, cefotaxime, 30 μg; KAN, kanamycin, 30 μg; CEP, cephalothin, 30 μg; STR, streptomycin, 10 μg; SXT, trimethoprim/sulfamethoxazole, 1.25/23.75 μg; TET, tetracycline, 30 μg; TMP, trimethoprim, 5 μg.
